# Analysis of the *Pvu*II and *Xba*I polymorphisms in the estrogen receptor alpha gene in girls with central precocious puberty: a pilot study

**DOI:** 10.1186/s12881-018-0577-x

**Published:** 2018-05-25

**Authors:** José Maria Soares-Jr, Felisbela Soares de Holanda, Cézar Noboru Matsuzaki, Isabel Cristina Esposito Sorpreso, Eduardo Carvalho de Arruda Veiga, Luiz Carlos de Abreu, Kátia Cândido Carvalho, Edmund Chada Baracat

**Affiliations:** 1 Disciplina de Ginecologia, Departamento de Obstetrícia e Ginecologia, Hospital das Clínicas, Faculdade de Medicina da USP, Av. Dr. Eneas de Carvalho Aguiar, 255 - Predio do Instituto Central, 10 andar, sala 10167, São Paulo, 05403-000 Brazil; 2 Department of Gynecology, UNIFESP, São Paulo, Brazil; 3 Discipline of Science Design and Writing - Medical School of ABC, São Paulo, Brazil

**Keywords:** Puberty, Central precocious puberty, Gene of the estrogen receptor α (ESR1), *Pvu*II polymorphism, *Xba*I polymorphism

## Abstract

**Background:**

Precocious puberty (PP) is defined as premature pubertal development. Its consequences surpass the physical evidence of sexual maturity with the premature epiphyseal closure of the long bones and the reduction of adult stature by varied degrees. Central PP is characteristically dependent on GnRH and most of its causes are not completely known. Altered estrogen action is also believed to be involved in the genesis of PP. In fact, estrogen receptor alpha (Rea) gene polymorphisms may be associated with early age at menarche. The objective of this study was to investigate the relationship between Reα gene polymorphisms (PvuII and XbaI) and the occurrence of central PP.

**Methods:**

A total of 73 girls with central PP and 101 girls with normal pubertal maturation were evaluated. Both groups were genotyped for the PvuII (T/C) and XbaI (A/G) polymorphisms in the Reα gene.

**Results:**

The frequency distribution of the XbaI (*p* = 0.28) and of the PvuII (*p* = 0.12) genotypes, as well as the XbaI and PvuII allelic variants (*p* = 0.23 and *p* = 0.86, respectively), did not differ between the groups.

**Conclusion:**

The PvuII and XbaI Rea gene polymorphisms do not appear to be related to development of central PP.

**Electronic supplementary material:**

The online version of this article (10.1186/s12881-018-0577-x) contains supplementary material, which is available to authorized users.

## Background

Precocious puberty (PP) is characterized by premature pubertal development, that is, by the onset of puberty before 8 years of age. Such precociousness surpasses the physical evidence of sexual maturity with the premature epiphyseal closure of the long bones and the reduction of adult stature by varied degrees. The incidence is approximately 20/10,000 in that age group [1].

In 50% of patients with PP of central origin, onset is between 6 to 7 years [2]. Central precocious puberty (CPP) occurs more frequently in girls than in boys (approximately 20:1), and about 90% of the female cases are idiopathic (unknown cause) or constitutional (family heritage) [1, 3, 4].

The cause of most CPP cases is still unknown. The main determinant of the onset of puberty seems to be genetic. In studies with twins, monozygotic twins were shown to be in closer agreement with respect to bone age, age at menarche, increased height, and pubertal development than were dizygotic twins [5].

There are several hypotheses for the onset of PP involving neurotransmitters, peptides, and hormones [6–8]. However, little is known about the influence of estrogen and its receptor on the premature activation of the hypothalamic-hypophyseal-ovarian axis. In fact, estrogen administration to immature female rats 5 to 10 days old led to pulsatile secretion of GnRH, typical of the process of hypothalamic maturation. The secretion, in turn, led to symptoms of PP [9]. Perhaps estrogen is not only a consequence of the process of puberty onset in humans, but also a contributory factor in the maturation of the hypothalamic-hypophyseal-ovarian (HHO) axis via its receptor. This possibility has spurred a great deal of research [10–12]. Polymorphisms have been reported in intron 1 of the estrogen receptor gene, especially *Pvu*II (REα-T397C) and *Xba*I (REα-A351G) [13, 14]. Also, these polymorphisms are related to high risk of breast cancer and early menarche [15–17]. In addition, those polymorphisms seem to be the most prevalent and might interfere with the estrogen effect. The meta-analysis of Korean and China studies suggests that ESR1 XbaI and PvuII polymorphisms are associated with precocious puberty susceptibility [18]. However, there has not described these polymorphisms on the precocious puberty in Brazilian population yet. Therefore, our study aimed at determining whether the polymorphisms which influence estrogen receptor activity were present at elevated frequency in girls with PP.

## Methods

### Characteristics of the study population

This study was conducted at the Gynecology Division of Childhood and Adolescence, Discipline of Gynecological Endocrinology, Gynecology Department, São Paulo Federal University – Paulista School of Medicine (UNIFESP-EPM). In the pre-selection phase, a medical history was taken of the girls with the help of their guardians, including clinical data, personal history, and family history. The girls underwent a clinical and a gynecological examination. Inclusion criteria for the study group (73 girls) consisted of a previous or current diagnosis of PPP corroborated by physical examination or medical reports, and for the control group (104 girls), normal pubertal development after 8 years of age. Exclusion criteria were the presence of ovarian or adrenal neoplasms, the McCune-Albright syndrome, changes in the central nervous system (tumors, congenital malformations, infections, and traumas), laboratory or clinical assessment of hypothyroidism, use of hormones prior to diagnosis and use of psychotropic drugs.

### Extraction of genetic material and genotyping

DNA was extracted from peripheral blood leukocytes using standard procedures. The intron from the *ESR1* gene was amplified by PCR using 50 ng of the genomic DNA in a final reaction volume of 50 μl containing 5 μl of buffer, 0.2 mM dNTPs (from each of the four deoxyribonucleotides), 0.2 μM of each primer pair that spanned the polymorphic regions, 2 mM MgSO4, and 1 U Platinum® Taq DNA Polymerase High Fidelity (Invitrogen). The reaction took place in the VERITI (Apllied Biosystems) thermocycler, using the following cycle: preheating (94 °C for 2 min), denaturing for 40 cycles (94 °C for 60 s), annealing (50 °C for 90 s), extending (72 °C for 180 s), and extending one more final time (72 °C for 7 min). The primers used were sense 5′- CTG CCA CCC TAT CTG TAT CTT TTC CTA TTC TCC -3′ and antisense 5′- TCT TTC TCT GCC ACC CTG GCG TCG ATT ATC TGA -3′ [19].

After amplification, the PCR products were digested by the PCR-RFLP (polymerase chain reaction-restriction fragment length polymorphism) technique separately with the restriction enzymes, with recognition of the following sequence: 5′...CAG ↓ CTG...3′ and 5′...T ↓ CTAGA...3′, respectively for *Pvu*ll *and Xba*l in a final volume of 20 μl at 37 °C for 16 h, containing 6 U *Pvu*ll and 5 U *Xba*I (Promega), 2 μl enzyme buffer, 0.2 μl BSA (bovine serum albumin), and 17.3 μl PCR product. Fragment analysis was performed in 1% agarose gel stained with ethidium bromide, and the bands were separated and photographed under ultraviolet light. The PCR and RFLP conditions utilized followed the protocol of Souza et al. [16, 17]. The restriction enzymes and the base pair (bp) sizes for the normal and variant alleles are summarized in Table 1. We excluded three participants of control group due to problems with the blood collection. The final number is 101 girls in this group. We included the database in the supplemental files (Additional file 1).

**Table 1:** Polymorphisms, restriction enzymes, allelic variants, andsize of DNA fragment generated by PCR-RFLP

Polymorphisms	Enzymes	Allelic variant	Fragment size (bp)
REα − 351 A/G	*Xba*I	A (wild)	1374
		G (mutant)	936 + 438
REα −397 T/C	*Pvu*II	T(wild)	1374
		C (mutant)	931 + 443

### Statistical analysis

The data collection was followed by calculation of the mean and standard deviation of the mean. The results were shown as mean ± standard error of the mean. When two groups were compared and the variables were qualitative, the Fischer exact test was used, and when the variables were quantitative, the Student *t* test was applied. For polymorphisms, the Hardy-Weinberg (H-W) equilibrium was calculated. The Power Calculation of 80% was 70 patients per group based on the early menarche age frequency of Pvull/Xbal and high risk of breast cancer [16, 17]. The analyses were carried out with the SPSS version 16.0 software. All of the tests had the rejection level of the null hypothesis set at 0.05 or 5% (*p* < 0.05).

## Results

The main features of the study population and the frequencies of the different genotypes are displayed in Table 2. There was no significant difference between the control group (101 girls) and the study group (73 girls) with respect to the proportion of White and non-White girls (*p* = 0.69), the percentage of ancestors with CPP, or the mother’s age at menarche (control group: 149.1 ± 16.78 months and study group: 143.1 ± 174.23 months). Both groups had the same mean of body mass index (BMI) (control group: 20.9 ± 3.67 and study group: 21.2 ± 4.99); however, the percentage of obese patients (BMI > 30) was higher in the control group (6 cases) than in the study group (2 cases).

**Table 2:** Mean, standard deviation, and frequency distribution (%) of the participants’ characteristics

	Control group (*n* = 101)	Study group (*n* = 73)	*p*
Ethnicity			
Caucasian - *n* (%)	25 (24.75)	16 (21.9)	0.69
Non-Caucasian - *n*, %	75 (74.25)	57 (78.1)	
Family history^a^ - *n*, %	19 (18.81)	18 (24.65)	0.34
Mother’s menarche (months) - mean ± SD	149.1 ± 16.78	143.1 ± 17.23	0.43
BMI - mean ± SD	20.9 ± 3.67	21.2 ± 4.99	0.85

^a^family history of precocious puberty, *n* number of participants, *SD* standard deviation

There was no difference between the two groups in the distribution of the *Xba*I (*p* = 0.28) or the *Pvu*II (*p* = 0.12) genotypes, nor in the frequency of the *Xba*I (*p* = 0.23) or *Pvu*II (*p* = 0.86) allelic variants (Additional file 1, Tables 3 and 4).

**Table 3:** Frequency distribution of the genotypes and Alleles of the *Xba*I polymorphism in patients with precocious puberty and normal puberty

Genotypes^1^	Control group (*n* = 101)*	Study group (*n* = 73)
AA - *n* (%)	15 (14.85)	12 (16.40)
AG - *n* (%)	69 (68,32)	33 (45.20)
GG - *n* (%)	17 (16.83)	28 (38.40)
Alleles^2^		
A – n** (%)	84 (49.41)	45 (42.45)
G – n** (%)	86 (50.59)	61 (57.55)
Total – n** (%)	170 (100)	106 (100)

^1^*p* = 0.28, *n* number of participants, * = control group is not consistent with Hardy-Weinberg equilibrium; ^2^*p* = 0.23; n** = number of participants with specific alleles

**Table 4:** Frequency distribution of the genotypes of the *Pvu*II polymorphism in patients with precocious puberty and normal puberty

Genotypes^1^	Control group (*n* = 101)	Study group (*n* = 73)
TT – *n* (%)	22 (21.78)	10 (13.69)
TC – *n* (%)	53 (52.48)	46 (63.02)
CC – *n* (%)	26 (25.74)	17 (23.29)
Alleles^2^		
T - n** (%)	75 (48.70)	56 (47.06)
C - n** (%)	79 (51.30)	63 (52.94)
Total - n** (%)	154 (100)	119 (100)

^**1**^*p* = 0.12; *n* number of participants; ^2^*p* = 0.86; n** = number of participants with specific alleles

The heterologous genotypes, A,G and T,C for Xbal and Pvull, respectively, were the most frequent genotypes detected in both the study and control groups. The most frequent allele for the *Xba*l polymorphism in each group was the G allele, which was not associated with an increased risk (OR:0.86 [95% CI = 0.51; 156]) for PP (*p* = 0.23). The most frequent allele for the *Pvu*II polymorphism in each group was the C allele, which was similar in both groups. The control group of Xbal was not consistent with with Hardy-Weinberg equilibrium.

The estimating haplotypes of both polymorphisms and their frequency differences of these between control group and study group are on Table 5.

**Table 5:** Frequency distribution of the genotypes of the association between *Pvu*II and Xbal polymorphisms in patients with precocious puberty and normal puberty

Genotypes associations	Control group (*n* = 101)				Study group (*n* = 73)			
	AA + TT	AA + TC	AA + CC	total	AA + TT	AA + TC	AA + CC	total
	4	7	4	15	0	11	1	12
	AG + TT	AG + TC	AG + CC		AG + TT	AG + TC	AG + CC	
	14	38	17	69	5	23	5	33
	GG + TT	GG + TC	GG + CC		GG + TT	GG + TC	GG + CC	
	4	8	5	17	5	12	11	28
Total	22	53	26	101	10	46	17	73

No statistical differences between Control and Study groups

**Fig. 1:**
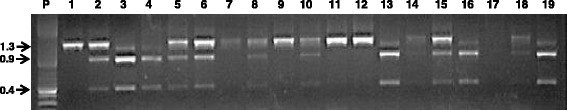
Representative gel of the polymorphism pattern resulting from the restriction with *Xba*I of amplified samples for the estrogen receptor alpha. Samples with one band only indicate a wild profile (~ 1300 pb, eg., lane 12), whereas samples with three bands (~ 1300, ~ 900, and ~400pb, eg., lanes 2 and 5) and with two bands (~ 900 and ~400pb, eg., lanes 3 and 4) indicate the heterozygous and homozygous profiles for mutation, respectively. The arrows point to the migration of each of the expected fragments. P, molecular weight standard (100 pb step ladder – Promega)

**Fig. 2:**
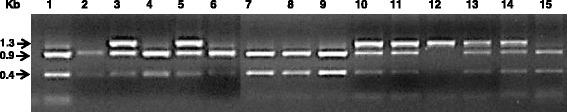
Representative gel of the polymorphism pattern resulting from the restriction with *Pvu*IIof amplified samples for the estrogen receptor alpha. Samples with one band only indicate a wild profile (~ 1300 pb, eg., lane 12), whereas samples with three bands (~ 1300, ~ 900, and ~400pb, eg., lanes 3 and 5) and with two bands (~ 900 and ~400pb, eg., lanes 7 and 9) indicate the heterozygous and homozygous profiles for mutation, respectively. The arrows point to the migration of each of the expected fragments

Figures 1 and 2 show the PCR-RFLP reactions for *Xba*l and *Pvu*II in a representative sample of the study participants.

## Discussion

It is still unclear what causes central precocious puberty [20]. There is an intense, ongoing debate about the involved mechanisms. Each individual’s genetic susceptibility, including polymorphisms, and the interaction of environmental and nutritional factors, are deemed important factors at puberty onset [21]. However, so far no consensus has been reached as to which of these determinants is the most important. This point should be emphasized to make it obvious why further research is needed. Our study aimed at evaluating the relationship between precocious puberty and estrogen receptor polymorphisms in Brazilian population. However, no association was found.

Epidemiologic studies report that many patients with central precocious puberty had a family history positive for this condition [22], thus suggesting a genetic inheritance. In our study, those with a family history of precocious puberty were only at a 2% greater risk of developing precocious puberty than were the girls who had no such history. It may be that the small number of girls with a positive family history in our study influenced the negative findings.

Most of the studies assessing the influence of genetic factors on puberty onset have attempted to correlate them with central factors [7, 23–25]. Few studies have evaluated the peripheral component as the trigger for precocious puberty [9]. According to Gorai et al. [10], estrogen is possibly the determinant in the maturation of the HHO axis, but we did not found the association of estrogen receptor polymorphism and central PP.

In different studies, the Reα gene polymorphisms have been associated with several pathological conditions, such as breast cancer, prostate cancer, osteoporosis, endometriosis, and cardiovascular diseases [26–28]. Parl et al. [29] found that the recessive mutant genotype of the *Pvu*II polymorphism was more abundant in women with breast cancer, which is related to estrogen action.

However, few researchers have assessed these genes in connection with age at menarche. Stavrou et al. [19] found that individuals with a wild-type genotype seemed to have a slightly delayed age at menarche. In our study, the same genotypes were also associated with an age delay at menarche. Nevertheless, further studies are needed to produce conclusive results with respect to an association between the *Pvu*II polymorphism and puberty onset. A delayed menarche is known to be a protection factor against breast cancer and endometrial cancer, because it reduces the exposure time of these tissues to estrogen.

Lee et al. [30] failed to show a connection between the aforementioned polymorphisms and puberty onset and progression in Korean girls, notwithstanding the more homogeneous population sample. In addition, Luo et al. [18] described a metanalysis with studies with Korean and Chinese population and found that ESR1 XbaI and PvuII polymorphisms are associated with precocious puberty susceptibility. However, this revision there are some bias, such some number of manuscript (*n* = 3) and small samples in the studies. We did not found manuscripts with European and South America population to compare our data. Besides, some authors suggest a prevalence of precocious puberty among girls of African descent [21]. Our results, however, drawn from a mixed racial population characteristic of Brazil, did not show a significant association between both polymorphism and central precocious puberty.

Research into the mechanisms leading to precocious puberty is necessary to prescribe an early and more efficacious treatment. In the last decades, many researchers have focused on the discovery of genetic markers for an early diagnosis as well as on the development of new and more effective therapies based on the biomarkers, with the possible outcome of improved prognosis, that is, an increase in height for the patient. Even though there was no significant difference between the groups in our study, perhaps due to the small sample or lack of subjects with a family history of PP, continuing studies with these polymorphisms are still a necessity before an association with this disorder can be rejected. It should be noted that the incidence of precocious puberty in the population is small, approximately 20/10,000 girls [1], making research slow and laborious. Therefore our study has some limitations, such as the small sample. Also, we found that the Xbal genotype distribution in control group was not consistent with Hardy-Weinberg equilibrium. Perhaps, a confirmation of genotyping method using a fraction of pregenotyped sample using a sequencing randomly and concordant between the techniques may decrease this problem.

## Conclusion

The development of precocious puberty is not related with estrogen receptor alpha gene polymorphisms (*Pvu*II and *Xba*I).

## Additional files

Additional file 1: Polymorphism data. Description of data: The XBAl and PvuII Polymorphism data of control and study (precocious puberty) were on the additional file. (XLSX 37 kb)

## Abbreviations

BMI: Body mass index
CPP: Central precocious puberty
ESR1: Gene of the estrogen receptor α
PP: Precocious puberty
*Pvu*II and *Xba*I: Reα gene polymorphisms
Rea: Estrogen receptor alpha
